# Cardiovascular Side Effects of Atomoxetine and Its Interactions with Inhibitors of the Cytochrome P450 System

**DOI:** 10.1155/2011/952584

**Published:** 2011-07-03

**Authors:** Pashtoon Murtaza Kasi, Rawad Mounzer, George H. Gleeson

**Affiliations:** ^1^PGY1, International Scholars Program, University of Pittsburgh Medical Center, Pittsburgh, PA 15213, USA; ^2^PGY3, International Scholars Program, University of Pittsburgh Medical Center, Pittsburgh, PA 15213, USA; ^3^University of Pittsburgh School of Medicine, Pittsburgh, PA 15261, USA

## Abstract

Attention deficit hyperactivity disorder (ADHD) is one of the most common neurobehavioral disorders of childhood and adolescence. Classically, stimulants have been used in the treatment of this condition. Atomoxetine (Strattera; Eli Lilly and Company) is a selective norepinephrine reuptake inhibitor (SNRI), one of the first medications in the nonstimulant class of medications that has been approved by the FDA for the treatment of ADHD. Atomoxetine is a phenoxypropylamine derivative and is structurally related to the antidepressant fluoxetine. The common side effects reported with the use of atomoxetine include mainly GI disturbances. Cardiovascular side effects are less commonly reported. The increase in the noradrenergic tone may explain some of the side effects noted with the use of this medication. Here, we present a case of a patient who presented with syncope, orthostatic hypotension, and tachycardia and discuss the various clinical implications based on the pharmacokinetics and pharmacodynamics of the drug.

## 1. Introduction

Attention deficit hyperactivity disorder (ADHD) is one of the most common neurobehavioral disorders of childhood and adolescence [1]. It is estimated to affect 5.29% of school-aged children worldwide and is a problem that can continue into adulthood, with significant burden of disease on the patient and the family's lives [2]. Classically, stimulants have been used in the treatment of this condition [3]. Atomoxetine (Strattera; Eli Lilly and Company) is a selective norepinephrine reuptake inhibitor (SNRI), one of the first medications in the nonstimulant class of medications that has been approved by the FDA for the treatment of attention deficit hyperactivity disorder [4]. “Atomoxetine is a phenoxypropylamine derivative and is structurally related to the antidepressant fluoxetine” [5]. The common side effects reported with the use of atomoxetine include “headache, abdominal pain, nausea, vomiting, decreased appetite, weight loss, irritability, insomnia and sedation” [6]. Cardiovascular side effects are less commonly reported. The increase in the noradrenergic tone may explain some of the side effects noted with the use of this medication. Here, we present a case of a patient who presented with syncope, orthostatic hypotension, and tachycardia and discuss the various clinical implications based on the pharmacokinetics and pharmacodynamics of the drug.

## 2. Case Report

A 26-years-old man with past medical history significant for mild mental retardation, obsessive compulsive disorder, and attention deficit hyperactivity disorder was admitted to our service after falling at his workplace after rising from a chair. It was a witnessed fall. Patient collapsed after rising from his chair after eating lunch at work. There was no urinary of fecal incontinence, no tongue biting, no jerky movements, and no loss of consciousness. There was no confusion or altered mental status afterwards. 

However, interestingly, the patient was tachycardic from 120 s to 140 s on presentation and was also noted to have orthostatic hypotension, despite IV hydration in the ED. Of note, patient's intake has always been good and rather he enjoys drinking plenty of fluids. The review of systems was negative, and the patient did not have any other signs or symptoms concerning any infection. Physical exam was significant only for mildly dilated pupils. 

Electrocardiogram (ECG) revealed sinus tachycardia, with no ST changes, and QTc interval was noted to be within the normal limits. Echocardiography showed a normal left ventricular size with mild hypertrophy with a low normal systolic function (ejection fraction of 50–55%). Segmental LV function was normal. Right ventricular size and function were normal, and no valvular abnormalities were noted. A head CT done in the emergency department also did not reveal any masses or lesions, and there were no fractures or hematomas. Thyroid studies were also within normal limits.

As per the patient's parent, who has been taking care of him for the past 26 years, the patient has never had these symptoms. The patient apparently has been on atomoxetine (Strattera; Eli Lilly and Company), for the last 6 years without any problems. When asked about any new changes or additions to his medications, the patient was recently started on fluoxetine (Prozac) couple of months ago to help him with the “tic” of picking at his face. 

The tachycardia, orthostatic hypotension, and the syncope were new and important. Given no organic problems to explain his symptoms, we reviewed the side effects profile of atomoxetine and any potential drug interactions with fluoxetine, since the symptoms temporally correlated with the onset of the new drug. Interestingly, as discussed below, fluoxetine does affect the metabolism of atomoxetine causing drug levels of the latter to rise by several folds. Following is an account of the cardiovascular side effects of atomoxetine (Strattera) with a discussion on clinical implications and ways forward.

## 3. Discussion

Table 1 outlines some of the common cardiovascular side effects noted with the use of atomoxetine. Increase in the heart rate and blood pressure appears to be dose dependent and the effects decrease with the cessation of therapy [8]. 

As noted in a recent review by Hammerness and colleagues, “serious cardiovascular side effects have not been noted in trials with therapeutic doses of atomoxetine” [4]. Usually, the increase in heart rate was estimated to be between 5 and 9 beats. However, in a report of 5 large placebo-controlled clinical trials of 612 patients, 3.6% of children/adolescents had a rise in their heart rate of at least 25 beats per minute or heart rate >110 [8]. 

With respect to orthostatic hypotension and syncope, “in child and adolescent trials, 0.2% (12/5596) of strattera-treated patients experienced orthostatic hypotension and 0.8% (46/5596) experienced syncope. In short-term child and adolescent-controlled trials, 1.8% (6/340) of strattera-treated patients experienced orthostatic hypotension compared with 0.5% (1/207) of placebo-treated patients. Syncope was not reported during short-term child and adolescent placebocontrolled ADHD trials” [7]. 

Moreover, from the clinical standpoint, there are 3 important considerations that we wanted to highlight in patients who are on long-term treatment with this drug. 

Firstly, the drug is mainly metabolized by the cytochrome P450 system (CYP2D6), and genetically people can have differential activity of the enzyme, hence, the terms “poor metabolizers” (PM) and “extensive metabolizers” (EM). It is estimated that approximately 7% of the Caucasian population may be poor metabolizers, leading to slow metabolism (5.2 versus 22 hours in PM versus EM) and higher drug levels (estimated 5 times higher) [9]. Genetic testing is available at various centers and may be considered in select patients who may need to be on long-term therapy with the medication or in individuals who start exhibiting serious side effects despite being on therapeutic doses.

Secondly, often at times, especially now with polypharmacy becoming increasingly common, potential drug interactions are easily forgotten. With the advent of newer drugs coming into the market, this aspect of clinical care would be of paramount importance. In this specific case, inhibitors of the cytochrome P450 (CYP2D6) (outlined in Table 2) can increase drug levels by several folds [10] and may account for the new onset of symptoms in a patient who previously was tolerating the medication without any problems. The pharmacokinetic profile of patients who are on drugs that inhibit the cytochrome P450 (CYP2D6) is seen to resemble that of patients who are “poor metabolizers” [10]. 

Thirdly, there have been case reports of QT interval prolongation with the use of this drug, which may lead to life-threatening arrythmias and may account for the cases of sudden cardiac death reported with the use of the drug. Based on these reports, in vitro studies on guinae pig cardiac myocytes and human embryonic kidney cells, Scherer and colleagues were able to identify a molecular basis for the side effect noted [5]. The SNRI atomoxetine apparently inhibits hERG-potassium channels, leading to prolongation of the QT interval. Though the effect is mild at therapeutic concentrations, this should be considered in patients who are poor metabolizers, are on inhibitors of the cytochrome system, or exhibit side effects related to drug overdose. ECGs should also be done periodically in patients on long-term use of this drug, and the drug should be avoided in patients with preexisting cardiac problems or conditions that may make them prone to arrhythmias [11]. 

In our patient, after discussion with the patient's psychiatrist, we discontinued patient's fluoxetine since it was not helping his symptoms of “tic” for which it was started earlier. With respect to his atomoxetine, we held it for a few days following which patient would be restarted on a lower dose for now with close followup and/or potentially other options [7]. Previous studies have also reported the cardiovascular side effects seemed to resolve with the discontinuation of the medication [6]. As per prescribing information from the manufacturer's website, the maximum adult daily dose recommended is not more than 100 mg per day, with dosage adjustment in patients with hepatic impairment and presence of a strong CYP2D6 inhibitor and patients known to be poor metabolizers. The addition of fluoxetine (20 mg) to our patient's medication regimen (100 mg daily) without adjustment of doses most likely led to supratherapeutic drug levels, explaining the side effects experienced by our patient. 

Patients who need to be on atomoxetine need to have frequent monitoring of their cardiovascular symptoms on their follow-up visits (measuring blood pressure and heart rate at each visit and EKG if there are any concerns), along with an eye on potential drug interactions before starting any new medication that might affect the metabolism of the drug. Studies into the molecular basis of the drug are revealing newer insights explaining the cardiovascular side effect profile of the medication.

## Figures and Tables

**Table 1:** Cardiovascular side effects of atomoxetine (Strattera) [1, 2, 5, 7].

*Common side effects*	
Increase in heart rate	
Sinus tachycardia	
Increase in systolic pressure especially in adults	
Increase in diastolic blood pressure especially in children	
Palpitations	

*Less common/case reports*	
Prolongation of QT interval	
Orthostatic hypotension	
Raynaud's phenomenon	
Syncope	
Chest pain	
Sudden death	

**Table 2:** Some of the commonly used drugs that inhibit the Cytochrome P450 (CYP2D6) system [3, 6, 7].

Fluoxetine
Paroxetine
Quinidine
Citalopram
Escitalopram
Bupropion
Sertraline
Chlorpromazine
Hydroxyzine
Clomipramine
